# Characterization and Correlation of Microbial Communities and Volatile Flavor Compounds in Cabbage Kimchi

**DOI:** 10.1002/fsn3.71042

**Published:** 2025-12-01

**Authors:** Aiguo Luo, Xin Guo, Wenjie Guan, Shengli Shi, Qi Sun, Bianfang Hu

**Affiliations:** ^1^ Department of Biological Science and Technology Jinzhong University Jinzhong China; ^2^ Shanxi Center of Technology Innovation for Compound Seasonings Jinzhong University Jinzhong China; ^3^ College of Life Sciences Shanxi Agricultural University Jinzhong China; ^4^ Jinzhong Normal College Jinzhong China

**Keywords:** correlation analysis, kimchi fermentation, microbial communities, volatile flavor compounds

## Abstract

In order to reveal the changes in microbial communities and volatile flavor compounds during the fermentation process of cabbage kimchi and the correlation between them, high‐throughput sequencing (NGS) and headspace solid‐phase microextraction gas chromatography–mass spectrometry (HS‐SPME/GC–MS) were used to study the microbial diversity and volatile flavor components of kimchi on Days 1, 3, 5, 7, 9, and 10 of fermentation. The correlation between microbial diversity and volatile flavor compounds was also analyzed in detail. The results showed that, in the microbial analysis of kimchi, 115 bacterial genera were identified, including *Lactiplantibacillus*, *Bacillus*, *Staphylococcus*, and *Tetragenococcus*, among others. The average relative abundance of *Lactiplantibacillus* was 23.44%, making it the most predominant bacterial genus in kimchi. Additionally, 176 fungal genera were identified, including *Membranomyces*, *Aureobasidium*, *Scedosporium*, *Neomicrosphaeropsis*, *Phallus*, and *Saccharomycopsis*, among others. *Membranomyces* had the highest average relative abundance at 85.63%, making it the most abundant fungal genus in kimchi. In the analysis of volatile flavor compounds in kimchi, 102 volatile compounds were detected, including methanethiol, linalool, and α‐pinene, etc. The average relative content of acetic acid was 6.76%, and the average relative content of dimethyl disulfide was 7.32%, making them the highest among the volatile flavor compounds detected. Alcohols and sulfur‐containing compounds were the main volatile flavor compounds. According to the correlation analysis using SPSS, 18 bacterial genera showed a strong positive correlation with volatile flavor compounds, and 15 fungal genera showed a strong positive correlation, with correlation coefficients > 0.9. The correlation analysis indicated that *Lactiplantibacillus*, *Bacillus*, *Staphylococcus*, and *Tetragenococcus* are the core bacterial genera responsible for producing key volatile flavor compounds in kimchi, while *Membranomyces*, *Aureobasidium*, *Scedosporium*, *Neomicrosphaeropsis*, *Phallus*, and *Saccharomycopsis* are the core fungal genera.

## Graphical abstract

This study integrated high‐throughput sequencing with HS‐SPME/GC‐MS to systematically investigate the dynamic interplay between microbial communities and volatile flavor compounds during cabbage kimchi fermentation. Results revealed 115 bacterial and 176 fungal genera at the genus level, with Lactiplantibacillus (mean relative abundance 23.44%) and Membranomyces (85.63%) being the predominant bacterial and fungal genera, respectively. A total of 102 volatile compounds were identified, predominantly alcohols and sulfur‐containing compounds. Pearson correlation analysis identified 18 bacterial and 15 fungal genera exhibiting strong positive correlations (*r* > 0.9) with key flavor compounds, pinpointing core functional microbiota, including *Lactiplantibacillus*, *Staphylococcus*, *Tetragenococcus*, *Membranomyces*, and *Aureobasidium*, as crucial drivers in the formation of kimchi's characteristic flavor profile.

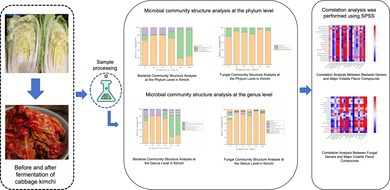

## 1 Introduction

Kimchi, a globally significant traditional fermented food, exhibits substantial and expanding market demand (Choi et al. 2024; Park 2021). In China alone, the kimchi industry reached a market size of 73.5 billion CNY in 2023, reflecting an annual growth rate of 5.9%. However, heterogeneous consumer preferences—shaped by demographic and regional factors—necessitate continuous innovation and product diversification within the sector (Zhang et al. 2016). The characteristic flavor profile of kimchi arises from complex microbial metabolic activities during fermentation, where the dynamics of microbial communities directly govern the production of key volatile flavor compounds (Xiao et al. 2021; Baek et al. 2020; Cha et al. 2024).

Research on kimchi fermentation has extensively explored microbial community structure, diversity, and flavor chemistry using advanced methodologies. High‐throughput sequencing studies have elucidated microbial succession during fermentation (Hong et al. 2021; Shen et al. 2021; Li, Lao, et al. 2024; Li, Liu, et al. 2024) and compared diversity between natural and inoculated processes (Lingjuan et al. 2023; Zhao et al. 2023). Concurrently, headspace solid‐phase microextraction coupled with gas chromatography–mass spectrometry (HS‐SPME/GC–MS) has emerged as a predominant technique for volatile compound analysis, enabling comprehensive profiling of complex flavor matrices (Guo et al. 2022; Seo et al. 2024). Recent investigations have further correlated microbial composition with physicochemical quality parameters in paocai (Yang et al. 2022; Huang et al. 2021; Lian et al. 2024) and demonstrated how substrate geometry modulates early‐stage microbial dynamics and metabolite flux (Choi et al. 2023; Joseph et al. 2024; Zhang et al. 2024).

Despite these advances, critical knowledge gaps persist regarding cabbage kimchi, the most commercially prominent variant. Specifically, studies comprehensively characterizing: (i) Temporal shifts in its microbial consortia across key fermentation stages, (ii) The full spectrum of its volatile flavor compounds, and crucially, (iii) Correlative and potentially causative relationships between microbial taxa and flavor metabolite production remain limited. While a recent longitudinal study (Bae et al. 2024) investigated microbial and metabolic profiles in relation to seasonal harvest timing, systematic longitudinal studies concurrently tracking microbial community evolution and volatile compound dynamics throughout fermentation to establish specific causative links between microbial taxa and flavor metabolites are still lacking.

This study addresses these gaps by analyzing cabbage kimchi samples collected at strategic timepoints (Days 1, 3, 5, 7, 9, and 10) throughout fermentation. We integrate next‐generation sequencing (NGS) for high‐resolution microbial community profiling with HS‐SPME/GC–MS for quantitative volatile flavor compound analysis. By correlating these datasets, we elucidate microbiome‐flavor interactions, providing a mechanistic basis for targeted fermentation control to engineer desirable flavor profiles in industrial kimchi production.

## 2 Materials and Methods

### 2.1 Materials and Reagents

Cabbage, garlic, Sichuan pepper, chili, ginger, salt, and white vinegar were purchased from a local Sam's Club supermarket.

E.Z.N.A. Mag‐Bind Soil DNA Kit was purchased from Omega Bio‐tek, USA. Qubit dsDNA HS Assay Kit was obtained from Thermo Fisher Scientific, USA. 2× Hieff Robust PCR Master Mix and Hieff NGS DNA Selection Beads were provided by Yeasen Biotechnology (Shanghai) Co. Ltd., China.

### 2.2 Instruments and Equipment

Pico‐21 Bench‐top Centrifuge (Thermo Fisher, USA); GL‐88B Vortex Mixer (Qilinbeier Instrument Manufacturing Co. Ltd., Haimen); DYYC Electrophoresis Power Supply (Liu Er Instrument Factory, Beijing); DYCZ‐21 Electrophoresis Tank (Liu Yi Instrument Factory, Beijing); FR‐1000 Gel Imaging System (Shanghai Furi Technology Co. Ltd.); ETC 811 PCR Thermocycler (Dongsheng Innovation Biotechnology Co. Ltd., China); Thermo Fisher Trace 1310 Gas Chromatograph‐Mass Spectrometer (Thermo Fisher, USA); 65 μm (PDMS/DVB) Extraction Fiber (Supelco, USA).

### 2.3 Preparation of Samples

Cabbage kimchi was prepared using a traditional kimchi‐making method. A 1000 g head of cabbage was washed, chopped, and drained of excess water before being placed in a fermentation jar. To the jar, 60 g of garlic, 40 g of chili, 40 g of ginger, and 15 g of Sichuan pepper were added. Additionally, 80 g of sugar and 40 g of salt were dissolved in water, boiled, cooled, and poured into the kimchi jar. The jar was sealed and left to ferment under natural conditions. Samples were taken on Days 1, 3, 5, 7, 9, and 10 of fermentation. The kimchi was thoroughly mixed before sampling, and the samples collected at each time point were used simultaneously for microbial community high‐throughput sequencing and volatile flavor compound analysis. The samples were then stored, and volatile flavor compounds in the kimchi were measured using HS‐SPME/GC–MS. Microbial communities in the kimchi were analyzed using high‐throughput sequencing technology. After characterization, the correlation between microbial communities and volatile flavor compounds was analyzed.

### 2.4 Analysis of Kimchi Microbial Community Structure

Microbial genomic DNA was extracted from kimchi samples using a commercial kit. Bacterial (16S rRNA V3‐V4 region) and fungal (ITS1 region) amplicons were generated via PCR using universal primers: 341F (5′‐CCTACGGGNGGCWGCAG‐3′)/805R (5′‐GACTACHVGGGTATCTAATCC‐3′) for bacteria and ITS1F (5′‐CTTGGTCATTTAGAGGAAGTAA‐3′)/ITS2R (5′‐GCTGCGTTCTTCATCGATGC‐3′) for fungi. The PCR amplification system was as follows: 10× PCR buffer 5 μL, dNTPs 2 μL, DNA template 10 ng, forward and reverse primers each at 0.5 μmol/L, Taq DNA polymerase 0.05 U, with the volume adjusted to 50 μL using double‐distilled water.

PCR amplification conditions for bacteria were: 95°C for 3 min for pre‐denaturation; then 95°C for 30 s for denaturation, 45°C for 30 s for annealing, and 72°C for 30 s for extension, repeated for 5 cycles; followed by 95°C for 30 s for denaturation, 55°C for 30 s for annealing, and 72°C for 30 s for extension, repeated for 20 cycles; and finally, 72°C for 5 min for final extension (Park et al. 2012).

PCR amplification conditions for fungi were: 94°C for 3 min for pre‐denaturation; then 94°C for 30 s for denaturation, 45°C for 20 s for annealing, and 65°C for 30 s for extension, repeated for 5 cycles; followed by 94°C for 20 s for denaturation, 45°C for 20 s for annealing, and 65°C for 30 s for extension, repeated for 20 cycles; and finally, 72°C for 5 min for final extension (Kang et al. 2024).

After gel purification of the PCR products, they were accurately quantified and sent for Illumina MiSeq high‐throughput sequencing at Shenggong Bioengineering (Shanghai) Co Ltd.

### 2.5 Determination of Volatile Flavor Compounds in Kimchi

Volatile flavor compounds in kimchi were measured using the HS‐SPME/GC–MS method (Luo et al. 2023). 10 g of kimchi slurry was placed into a 20 mL headspace vial, sealed, and equilibrated in a 40°C water bath for 30 min. The extraction fiber was then inserted into the vial, and the fiber was exposed to extract the volatile compounds for 40 min. After extraction, the fiber was retracted and removed from the vial, and the fiber was inserted into the GC injection port for analysis for 5 min. The extraction head was then conditioned for 20 min.

GC conditions: DB‐5 capillary column (30 m × 0.25 mm × 0.5 μm); carrier gas: He; column flow rate: 1.0 mL/min; non‐split injection; injection port temperature: 250°C; the temperature program was: initial temperature 35°C, hold for 5 min, increase at 7°C/min to 150°C, then at 5°C/min to 230°C, hold for 5 min.

MS conditions: Ion source temperature: 250°C; interface temperature: 230°C; ionization mode: EI (electron impact); electron energy: 70 eV; mass scan range: 40–550 amu.

### 2.6 Data Statistics

In this study, microbial community structure was analyzed using high‐throughput sequencing, while volatile flavor compounds were determined by HS‐SPME/GC–MS. Based on paired samples collected at the same fermentation time points (Days 1, 3, 5, 7, 9, and 10), the top 20 bacterial genera and top 20 fungal genera with the highest relative abundance were selected from the sequencing results. Pearson correlation analysis between these dominant microorganisms and the major volatile flavor compounds was performed using SPSS 24.0, and the results were visualized accordingly.

The bacterial 16S rDNA V3‐V4 region and fungal ITS1‐ITS2 region in the kimchi samples were sequenced using the Illumina MiSeq platform. After obtaining high‐quality sequences, OTU clustering and species annotation were performed. The α‐diversity indices were calculated using Origin 2021 software, and bar plots and box plots were generated to illustrate the diversity differences in the microbial communities.

The volatile flavor compounds in the kimchi were measured using HS‐SPME/GC–MS, which detected various esters, alcohols, aldehydes, and ketones. Each sample was measured in triplicate, and the average value was taken. Graphs were created using Excel 2019.

The top 20 fungal genera and 20 bacterial genera with the highest relative abundance from the high‐throughput sequencing results were selected. Pearson correlation coefficients were calculated using SPSS 24.0 software to analyze the relationships between these microorganisms and volatile flavor compounds, and the results were visualized.

## 3 Results and Analysis

### 3.1 Microbial α‐Diversity Analysis of Kimchi

Illumina MiSeq sequencing yielded effective bacterial sequence counts for cabbage kimchi samples ranging from 104,261 to 144,199, with observed OTUs ranging from 116 to 146. Effective fungal sequence counts ranged from 106,006 to 142,535, with observed OTUs ranging from 146 to 199. The microbial α‐diversity results are presented in Table 1.

**TABLE 1:** Microbial α‐diversity.

	Microbial time/days	Number of sequences	OTU index	Shannon index	Chao index	Ace index	Simpson index	Coverage
Bacteria	1	142539	144	1.063735	152.260870	154.883986	0.490911	0.999860
	3	144199	116	0.612469	139.882353	140.205739	0.672019	0.999799
	5	133729	119	0.612702	123.935484	129.221705	0.675737	0.999865
	7	117774	127	1.034605	139.000000	147.074794	0.540872	0.999796
	9	104261	132	1.452824	136.565217	140.652011	0.405336	0.999856
	10	119497	146	1.475990	153.965517	163.036504	0.376172	0.999816
Fungi	1	142535	199	1.860746	215.866667	217.113659	0.340453	0.999839
	3	116711	187	1.177196	241.375000	210.190226	0.516622	0.999743
	5	106006	173	1.164610	186.541667	192.831683	0.501722	0.999755
	7	141318	167	1.494859	204.800000	232.071942	0.451527	0.999802
	9	128732	146	0.876296	170.166667	174.668218	0.584302	0.999767
	10	130052	170	1.218743	214.000000	204.253948	0.514110	0.999746

The α‐diversity indices, including ACE, Chao, Shannon, and Simpson, were used to evaluate the richness and diversity of the bacterial communities in cabbage kimchi during fermentation (Wu et al. 2020). The ACE and Chao indices were used to measure the richness of microbial communities and were positively correlated with community richness. In contrast, the Shannon and Simpson indices were applied to assess the diversity level of specific communities. The Shannon index was positively correlated with the complexity of the microbial community, while the Simpson index was negatively correlated with the complexity of the microbial community (Luo et al. 2025). For bacteria, the continuous decline of Chao and ACE indices from Day 1 to 5 reflected microbial reduction driven by nutrient competition and acidification stress in the initial fermentation phase. Subsequent significant recovery from Day 7 onward—stabilizing on Day 9 and reaching peak values on Day 10—indicated community reconstruction dominated by acid‐tolerant microbiota (e.g., Lactiplantibacillus). The Shannon index initially decreased, then sharply increased before declining again after Day 7, while the Simpson index showed an inverse trend. The highest OTU count, maximum Shannon index, and minimum Simpson index observed on Day 10 confirmed the most abundant microbial species with optimal even distribution at this stage, marking stabilized bacterial community diversity by the end of fermentation.

The fungal ACE index continuously decreased to its lowest value on Day 9 before rebounding on Day 10, while the Chao index fluctuated downward to its Day 9 minimum then increased. This trend revealed fungi's heightened sensitivity to acid stress (optimal pH higher than bacteria), though the competitive advantage of acid‐adapted Membranomyces maintained its ecological niche. Shannon and Simpson indices demonstrated an overall increase in fungal diversity, with the Day 10 diversity surge potentially linked to secondary metabolism activation by bacterial metabolites. Notably, synchronous significant changes in bacterial and fungal community diversity after Day 9 marked a critical transition from primary metabolism (environmental adaptation) to secondary metabolism (flavor synthesis), in line with the traditional kimchi “flavor turning point” theory.

### 3.2 Microbial Community Structure of Kimchi

**FIGURE 1:**
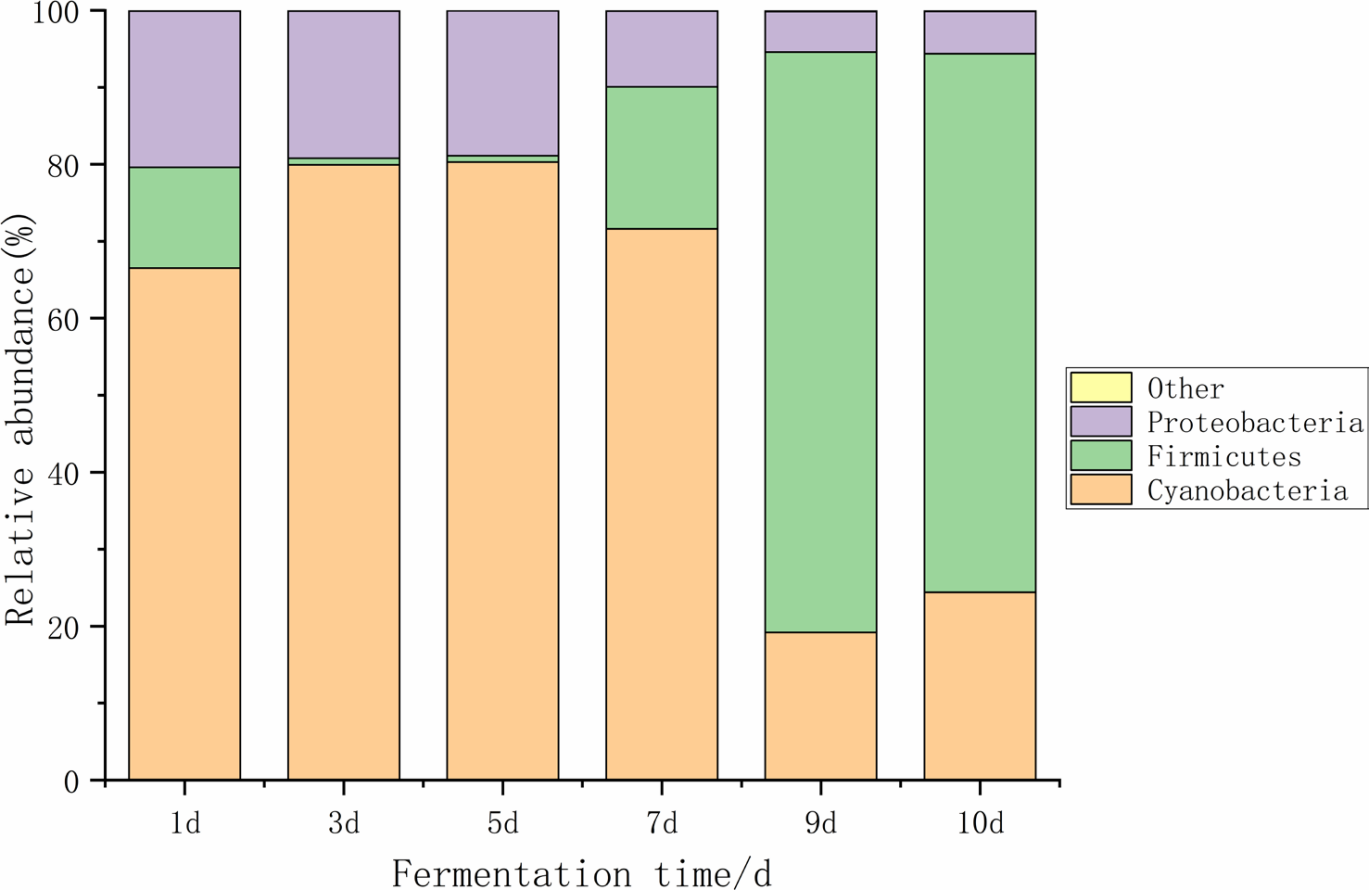
Bacterial community structure analysis at the phylum level in Kimchi.

As shown in Figure 1, the bacterial community structure at the phylum level in kimchi was analyzed. Through the annotation of bacterial OTUs, a total of 10 bacterial phyla were identified in the kimchi samples. Among them, three phyla had a relative abundance of 1% or more in at least one sample and were considered dominant phyla. The remaining non‐dominant phyla and unannotated OTUs were classified as “other.” These dominant phyla included Cyanobacteria, Firmicutes, and Proteobacteria, all of which had an average relative abundance > 1% in all samples. Cyanobacteria dominated the initial fermentation stage at 80.28% on Day 1, declined to a low of 19.21% by Day 5, and subsequently rebounded to over 50% in later stages, suggesting a possible origin from raw materials or the environment along with adaptive growth under acidic conditions. The abundance of Firmicutes increased significantly during the later phases, reaching 35% by Day 10, coinciding with the proliferation of lactic acid bacteria such as *Lactiplantibacillus*.

Throughout the fermentation process, Cyanobacteria was the most dominant phylum, with relative abundances ranging from 19.21% to 80.28%. This finding was consistent with the results from Li, Lao, et al. (2024) and Li, Liu, et al. (2024) who also identified Cyanobacteria as one of the key phyla in sauerkraut from Guizhou, where its presence contributed significantly to microbial diversity in regions such as QXN and YQ.

In the early stages of fermentation, the abundance of Firmicutes initially decreased, then gradually increased. On the other hand, Proteobacteria had a higher abundance in the early stages of fermentation, but its relative abundance exhibited a fluctuating decline as the fermentation process progressed.

**FIGURE 2:**
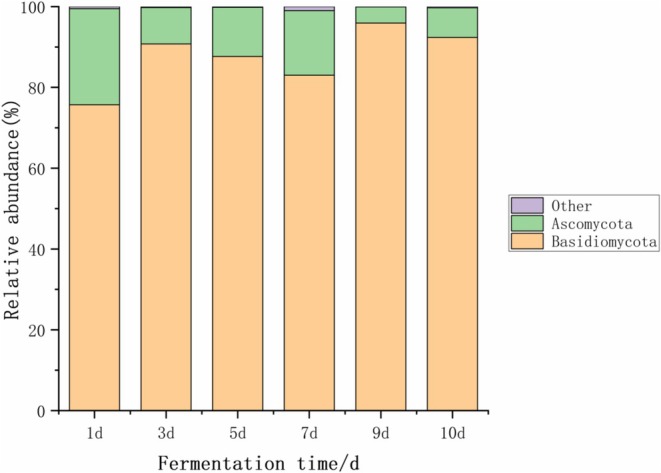
Fungal community structure analysis at the phylum level in Kimchi.

As shown in Figure 2, the fungal community structure at the phylum level in kimchi was analyzed. Through the annotation of fungal OTUs, a total of 6 fungal phyla were identified in the kimchi samples. Among all the tested samples, two phyla had an average relative abundance exceeding 1% and were therefore considered dominant phyla. These two dominant phyla were Basidiomycota and Ascomycota.

Throughout the fermentation process, Basidiomycota and Ascomycota showed significant dominance. Basidiomycota had the highest relative abundance, becoming the most dominant phylum. Its relative abundance fluctuated from the beginning to the end of fermentation and stabilized at 92.36% by the end of fermentation. The high abundance of Basidiomycota played a key role in driving changes in the active microbial community of kimchi. In contrast, Li, Lao, et al. (2024) and Li, Liu, et al. (2024) reported Candida (Ascomycota) and Sugiyamaella (Ascomycota) as the most abundant fungal genera in sauerkraut, indicating that while Ascomycota dominance is a common feature of vegetable fermentation, Basidiomycota prevalence in kimchi might reflect regional or process‐specific differences (Li, Lao, et al. 2024; Li, Liu, et al. 2024).

**FIGURE 3:**
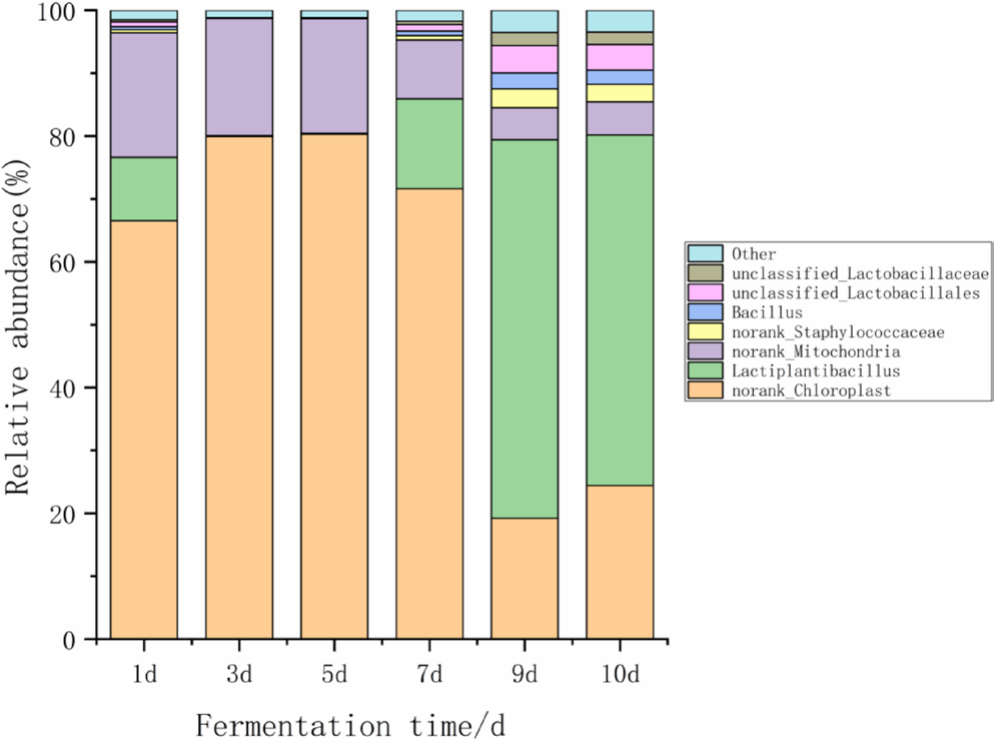
Bacterial community structure analysis at the genus level in Kimchi.

As shown in Figure 3, the bacterial community structure at the genus level in cabbage kimchi was analyzed. Through the analysis of bacterial genera in the kimchi samples, a total of 115 bacterial genera were identified. Among these bacterial genera, 8 were determined to be dominant genera, with a relative abundance of 1% or more in at least one sample. The proportion of *Lactiplantibacillus* increased steadily starting from Day 3 and reached a peak of 45.7% by Day 10, whereas *Staphylococcus* decreased from 22.3% on Day 1 to just 3.1% by the end of fermentation, indicating that lactic acid bacteria became the absolutely dominant microbial group in the later stages.

Throughout the fermentation process, *Lactiplantibacillus*, *Bacillus*, *Staphylococcus*, and *Tetragenococcus* were the dominant genera with higher average relative abundance across all samples. The high proportion of unclassified sequences, as well as those annotated as organelles (e.g., mitochondria and chloroplasts), is likely attributed to DNA derived from the cabbage plant material. This highlights the importance of rigorous bioinformatic processing to filter out non‐microbial sequences in food fermentation microbiome studies. After considering this, the remaining classified sequences suggest the microbial species in kimchi are complex.

**FIGURE 4:**
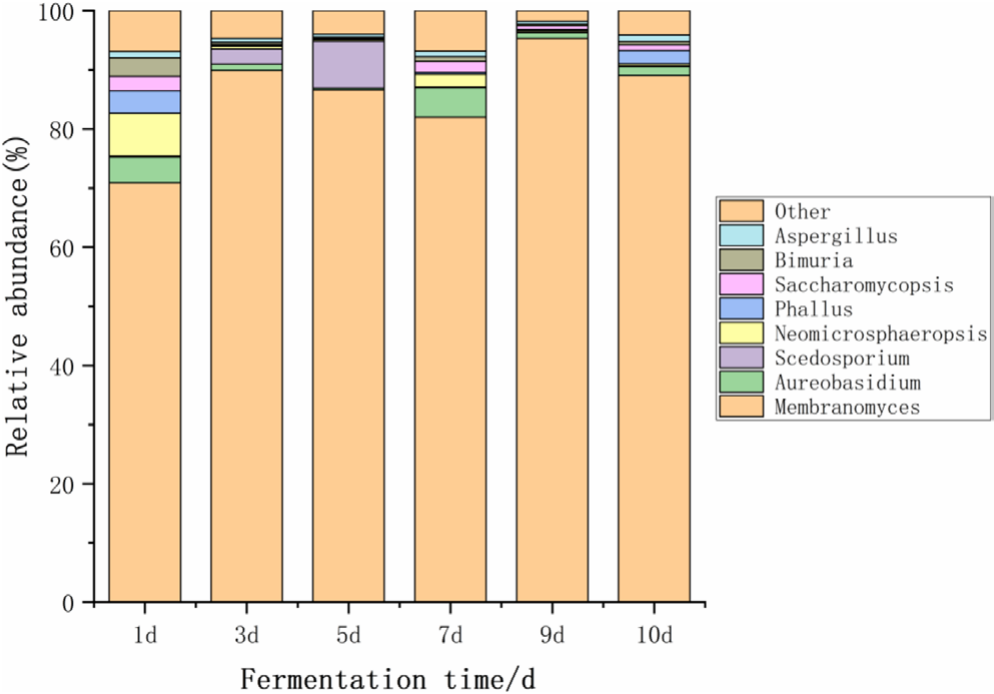
Fungal community structure analysis at the genus level in Kimchi.

As shown in Figure 4, the fungal community structure at the genus level in kimchi was analyzed. A total of 176 fungal genera were detected in the kimchi samples, of which 8 were considered dominant genera. Among all the samples, the dominant fungal genera with an average relative abundance > 1% included *Membranomyces*, *Aureobasidium*, *Scedosporium*, *Neomicrosphaeropsis*, *Phallus*, *Saccharomycopsis*, *Bimuria*, and *Aspergillus*.

### 3.3 Volatile Flavor Compound Measurement Results

The volatile flavor compounds in kimchi samples taken at Days 1, 3, 5, 7, 9, and 10 of fermentation were analyzed, and the types and contents of the volatile compounds are shown in Table 2, with the relative contents detailed in Table 3.

**TABLE 2:** Types and content of volatile flavor compounds in cabbage Kimchi.

Types of compounds	Time (days)											
	1		3		5		7		9		10	
	Number of species	Relative content/%	Number of species	Relative content/%	Number of species	Relative content/%	Number of species	Relative content/%	Number of species	Relative content/%	Number of species	Relative content/%
Alcohols	10	9.67	15	14.69	12	16.03	14	18.64	16	21.48	12	23.44
Hydrocarbons	13	9.56	11	7.32	11	8.2	10	6.96	9	6.37	5	2.18
Sulfur‐containing compounds	9	14.21	11	25.95	12	24.23	12	25.16	11	22.08	9	17.35
Aldehydes	16	18.43	16	13.62	15	12.49	14	10.24	15	9.43	13	8.02
Esters	8	8.31	7	6.59	7	4.05	6	4.63	7	3.46	4	7.31
Acids	1	2.34	2	5.22	2	6.62	3	9.05	3	7.81	4	6.33
Ketones	6	10.42	7	11.63	6	15.74	8	10.22	8	10.01	4	9.26
Others	4	7.32	4	7.01	4	6.95	3	8.44	3	8.41	3	7.92
Total	67		73		68		70		72		54	

**TABLE 3:** Relative content of volatile flavor compounds.

Compound name	Relative content/%					
	1 day	3 days	5 days	7 days	9 days	10 days
Acetic acid	1.94	5.1	10.83	7.75	7.62	7.3
Dimethyl disulfide	4.45	6.73	9.18	7.05	8.75	7.78
Methanethiol	5.06	8.26	5.06	0.91	0.14	0.08
Diglycolic anhydride	0.2	0.11	0.02			
Methyl allyl sulfide	0.37	0.61	2.18			
Diallyl sulfide	0.29	0.25	0.47			
1,3‐Dithiane	0.09	0.11	0.21	0.35	0.51	0.65
Toluene	5.28	1	0.02			
Myrcene	1.33	0.72	0.72	1.03	0.84	0.89
n‐Butanol	0.09	0.08	0.08			
Heptanol	0.11	0.03	0.03	0.04		
Undecane	0.32	0.02	0.09			
o‐Isopropyltoluene	0.13	0.41	0.48	0.43	0.36	0.33
p‐Tert‐butylbenzene	1.61					
α‐Phellandrene	0.05	0.05	0.07	0.05	0.07	
Ethylbenzene	1.1	0.11	0.03	0.05		
2,2‐Dimethylhexanone	0.02	0.02				
n‐Octanal	0.51	0.1	0.2	0.13	0.09	0.09
Hexanal	2.38	0.23	0.45			
Methyl propyl disulfide	0.03	0.14	0.11			
Allyl mercaptan	0.02	0.05	0.07	0.19		
N,N‐Dimethylthioacetamide	0.09	0.11				
Methyl heptenone	0.35	0.15	0.09	0.04	0.03	0.04
Allyl methyl disulfide	0.09	0.6	1.25	2.02	4.56	6.7
m‐Xylene	2.2	0.32	0.12			
2‐Methylpentanal	0.06	0.04	0.03	0.03		
2‐Phenylethyl isothiocyanate	0.04	0.06	0.06			
Benzaldehyde	0.23					
Heptanal	0.56	0.08	0.15	0.04	0.05	
Dimethyl trisulfide	0.25	4.81	4.3	3.22	1.6	2.45

A total of 102 volatile compounds were detected in the kimchi samples. The variation of these compounds and their contribution to the flavor of kimchi reflected the complex biochemical reactions and characteristics during the fermentation process. Sulfur‐containing compounds, such as dimethyl disulfide, reached their peak level of 25.95% on Day 3 and gradually decreased thereafter. In contrast, acidic compounds including acetic acid accumulated continuously, increasing to 9.05% by Day 7. The proportion of alcohols such as linalool rose steadily, reaching 23.44% by Day 10, a change associated with enhanced ethanol dehydrogenase activity of lactic acid bacteria. Aldehydes including hexanal were present at relatively high levels in the early fermentation stage, accounting for 18.43% on Day 1, but declined to 8.02% in the later phase due to microbial reduction, resulting in a diminished contribution to the overall flavor profile.

The main raw material of kimchi is cabbage, and its unique aroma mainly comes from sulfur‐containing compounds and alcohols. During fermentation, some volatile compounds in cabbage, such as terpenoid oils and methanethiol, are retained, greatly enriching the original aroma of the kimchi. Additionally, sulfur‐containing compounds have a distinct garlic‐like pungency (Ariga and Seki 2006), and their levels showed a trend of increasing initially and then decreasing. Particularly, the decline of methanethiol during the early stages of fermentation may have been due to its oxidation into more complex dimethyl compounds (Landaud et al. 2008). Similar patterns of sulfur compound dynamics were reported in Taizhou pickles, where early mustard‐derived isothiocyanates decreased over time, accompanied by increasing dimethyl disulfide and dimethyl trisulfide levels, highlighting the shared importance of sulfur compounds in Chinese fermented vegetables (Wang et al. 2025).

Alcoholic compounds consistently occupied a significant proportion during the fermentation cycle, especially linalool, α‐pinene, and eucalyptol. These compounds not only retained the characteristic aroma inherited from the raw material but also generated different aromatic compounds through microbial metabolism during fermentation. For example, the increase in 4‐terpineol could have been the result of lactic acid bacterial metabolism or adjunct ingredients, diverging from Seo et al. (2024) pure cabbage system. Consistent with our findings, the Taizhou pickle study also demonstrated that alcohol levels increased significantly during fermentation, particularly 1‐butanol and 2‐butanol, contributing to alcoholic, floral, and fruity aromas, which aligned with the observed rise in alcoholic notes in kimchi fermentation (Wang et al. 2025).

In terms of aldehydes, compounds such as hexanal, heptanal, and nonanal, which have a fresh aroma, were detected in the early fermentation stages from the cabbage raw material. However, as fermentation progressed, their content decreased, possibly due to microbial metabolism. This suggested that although aldehydes are present in the early stages of fermentation, they contributed minimally to the final product's aroma.

Acidic compounds continuously increased during fermentation, particularly acetic acid. Acetic acid helps regulate the pH of kimchi, enhances the sourness, and improves the overall flavor (Kang et al. 2024). The sustained increase in acetic acid was due to the metabolic byproducts of heterofermentative lactic acid bacteria during fermentation. Furthermore, acidic compounds can react with alcohols to form esters, adding complexity to the kimchi's aroma (Cha et al. 1998).

Esters in kimchi typically presented a rich, spicy aroma, with sulfur‐containing esters being the most prominent. These compounds contributed a strong spicy aroma that masked the sweet or fruity aromas of other esters, making them the dominant flavor profile of kimchi. While Seo et al. (2024) observed ethyl 2‐methylbutanoate accumulation confirming esterification, higher ester diversity in our study suggested enhanced reactions in complex fermentation matrices.

Hydrocarbons included alkanes and alkenes. Although alkanes contributed minimally to the aroma, alkenes made a contribution due to their distinctive aroma. For example, limonene remained relatively stable during fermentation, imparting a lemon‐like fresh scent to kimchi. However, in general, the concentration of hydrocarbons decreased, likely because unstable hydrocarbons were oxidized or reacted to form other types of compounds during fermentation. Although hydrocarbons were less discussed in Guizhou sauerkraut and Taizhou pickle studies, this stability of limonene mirrored findings in other fermented vegetable systems, where monoterpenes persisted to provide fresh citrusy notes (Wang et al. 2025).

Overall, the unique flavor of kimchi was formed by the combination of various volatile compounds, with different types of compounds increasing or decreasing as fermentation progresses, resulting in kimchi's distinctive and complex aroma.

### 3.4 Correlation Analysis Between Microorganisms and Volatile Flavor Compounds

The Pearson correlation coefficient was calculated and visualized for the top 20 volatile flavor compounds by relative content, and the top 20 bacterial genera and top 20 fungal genera by relative abundance.

**FIGURE 5:**
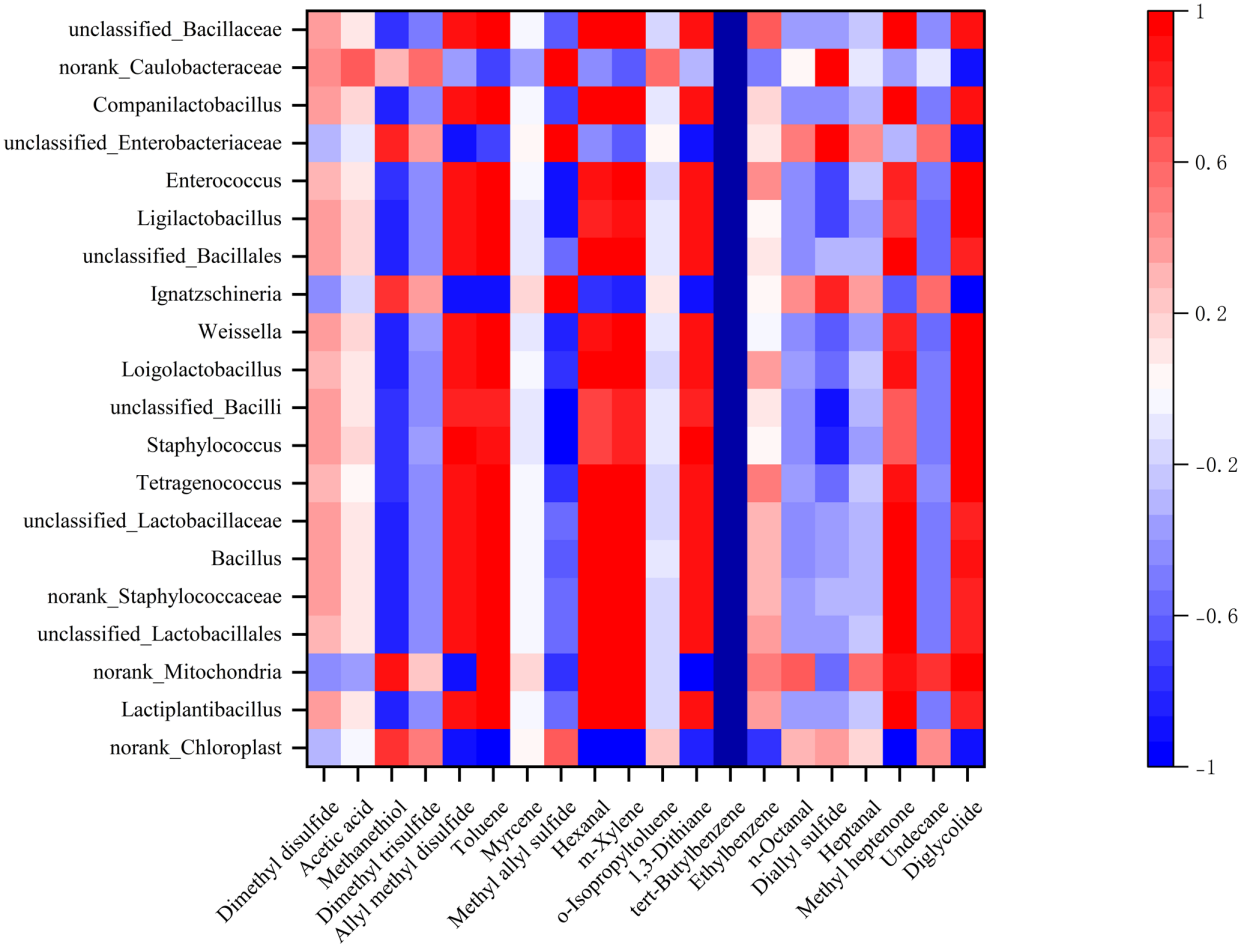
Correlation analysis between bacterial genera and major volatile flavor compounds.

The correlation analysis between bacterial genera and major volatile flavor compounds is shown in Figure 5. It could be observed that there was a significant correlation between different bacterial genera and volatile flavor compounds during kimchi fermentation. *Lactiplantibacillus* showed a strong correlation with several flavor compounds, especially with hexanal (0.996) and diglycolic anhydride (0.864), demonstrating a very strong positive correlation. In contrast, it shows a negative correlation with methanethiol (−0.820) and dimethyl trisulfide (−0.459). This suggested that *Lactiplantibacillus* may have played a promoting role in the production of some flavor compounds but may have had an inhibitory effect in others.

*Staphylococcus* displayed a strong positive correlation with 1,3‐dithiolane (0.934) and octanal (0.848), while showing a strong negative correlation with allyl methyl disulfide (−0.965). This implied that *Staphylococcus* may have played an important role in the production or consumption of specific compounds during the fermentation process of kimchi.

*Tetragenococcus* demonstrated a significant positive correlation with diglycolic anhydride (0.965) and 1,3‐dithiolane (0.899), while showing a negative correlation with allyl methyl disulfide (−0.786). This indicated that *Tetragenococcus* may have had a considerable influence on the concentration changes of these volatile flavor compounds during fermentation.

*Ignatzschineria* showed opposing correlations with several flavor compounds, such as a positive correlation with methanethiol (0.777) and a negative correlation with allyl methyl disulfide (−0.916). This suggested that *Ignatzschineria* may have had a complex role in the formation of kimchi's flavor.

**FIGURE 6:**
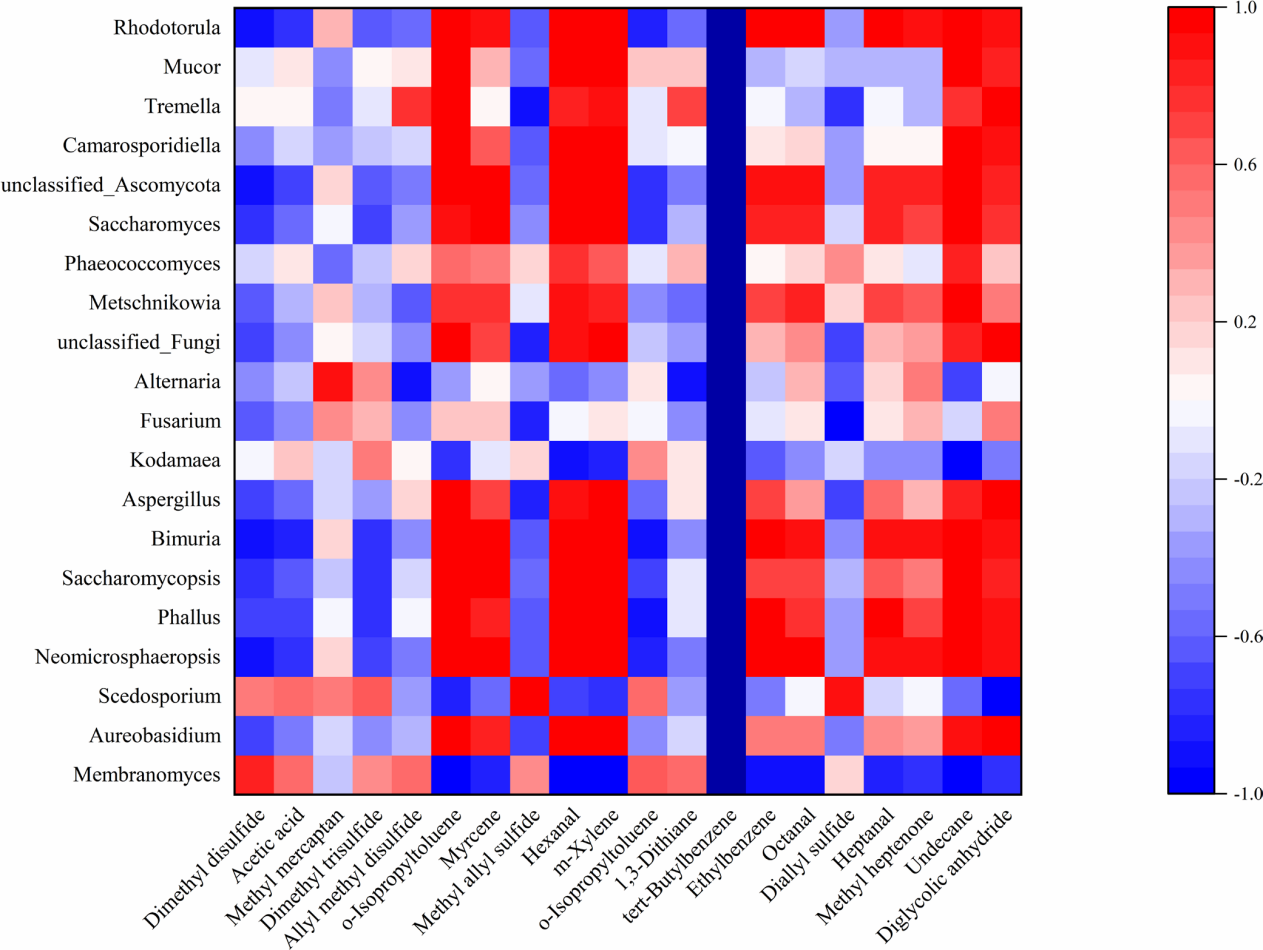
Correlation analysis between fungal genera and major volatile flavor compounds.

The correlation analysis between fungal genera and major volatile flavor compounds is shown in Figure 6. The correlation results indicated a strong relationship between the volatile flavor compounds of kimchi and different fungal genera. Specifically, *Membranomyces* showed a positive correlation with flavor compounds such as dimethyl disulfide, allyl methyl disulfide, and toluene, suggesting that this fungal genus may have played a significant role in the fermentation process of kimchi. *Aureobasidium* exhibited a high positive correlation with flavor compounds like toluene, laurene, and hexanal, indicating its potential substantial contribution to the specific flavor profile of kimchi. *Scedosporium* was positively correlated with dimethyl trisulfide, methyl allyl sulfide, and 1,3‐dithiolane, implying that it may have contributed to the formation of unique flavors during kimchi fermentation.

In addition, other fungal genera showed correlations with specific flavor compounds, such as *Neomicrosphaeropsis* with toluene and *Phallus* with methanethiol. These results suggested that different fungal genera may have influenced the flavor characteristics of kimchi through the release of their metabolic products during fermentation.

The observed strong correlations (|*r*| > 0.9) between specific microbial genera and flavor compounds reflected their potential metabolic roles in flavor formation. For instance, *Lactiplantibacillus*'s positive correlation with hexanal (0.996) aligned with its known capacity for lipid oxidation, which generates aldehydes from fatty acid precursors. Conversely, its negative correlation with methanethiol (−0.820) suggested potential degradation or transformation of sulfur compounds. Staphylococcus's positive correlation with 1,3‐dithiolane (0.934) indicated possible involvement in sulfur metabolism, contributing to pungent notes. Fungal genus *Membranomyces* showed significant positive correlations with dimethyl disulfide and allyl methyl disulfide—key compounds for garlic‐like aroma—implying its enzymatic role in sulfur compound biosynthesis.

These interactions highlighted how microbial consortia collectively drove the dynamic flavor evolution in kimchi, where bacterial activities dominated acid/alcohol production while fungi modulated sulfurous notes.

Through time‐series analysis, this study revealed a two‐phase pattern in the fermentation of cabbage kimchi. The first phase, from Days 1 to 5, was dominated by Cyanobacteria, Staphylococcus, and aldehydes, during which the microbial community underwent acidification adaptation and competitive reduction. The second phase, from Days 7 to 10, was characterized by the emergence of acid‐tolerant genera such as Lactiplantibacillus and fungi including Membranomyces as the core functional microbiota, driving the accumulation of acetic acid, alcohols, and sulfur‐containing compounds that contributed to the characteristic flavor profile. This dynamic process validated the existence of a flavor turning point and provided a theoretical basis for targeted regulation of the final fermentation flavor. Therefore, by considering these correlation results comprehensively, a better understanding could be gained of the impact of microbial communities during kimchi fermentation on the contribution to its flavor profile.

## 4 Conclusion

This study demonstrates that bacterial communities exhibit higher richness and diversity than fungi during cabbage kimchi fermentation. *Lactiplantibacillus*, *Bacillus*, *Staphylococcus*, and *Tetragenococcus* are the dominant bacterial genera, while *Membranomyces*, *Aureobasidium*, and *Saccharomycopsis* are the dominant fungal genera. These microorganisms have been identified as the core functional microbiota during the kimchi fermentation process. Volatile compound analysis revealed continuous increases in acids, alcohols, and sulfur‐containing compounds, while hydrocarbons, aldehydes, and ketones decreased. The key correlation analysis indicated that *Lactiplantibacillus*, *Staphylococcus*, *Tetragenococcus*, and *Membranomyces*, *Aureobasidium*, and *Saccharomycopsis* are the primary drivers of flavor compound formation.

## Conflicts of Interest

The authors declare no conflicts of interest.

## Data Availability

The data supporting the findings of this study are available from the corresponding author, Aiguo Luo, upon reasonable request.
